# Comparison of ethanol production from corn cobs and switchgrass following a pyrolysis-based biorefinery approach

**DOI:** 10.1186/s13068-016-0661-4

**Published:** 2016-11-09

**Authors:** Luis Luque, Stijn Oudenhoven, Roel Westerhof, Guus van Rossum, Franco Berruti, Sascha Kersten, Lars Rehmann

**Affiliations:** 1 Department of Chemical and Biochemical Engineering, University of Western Ontario, 1151 Richmond Street, London, ON Canada; 2 Sustainable Process Technology, Faculty of Science and Technology, University of Twente, P.O. Box 217, 7500 AE Enschede, The Netherlands; 3 Shell Global Solutions International BV, P.O. Box 38000, 1030 BN Amsterdam, The Netherlands; 4 Institute for Chemicals and Fuels from Alternative Resources, University of Western Ontario, 22312 Wonderland Road, Ilderton, ON Canada

**Keywords:** Biorefinery, Pyrolysis, Lignocellulose, Corn cobs, Switchgrass, Ethanol, Inhibition

## Abstract

**Background:**

One of the main obstacles in lignocellulosic ethanol production is the necessity of pretreatment and fractionation of the biomass feedstocks to produce sufficiently pure fermentable carbohydrates. In addition, the by-products (hemicellulose and lignin fraction) are of low value, when compared to dried distillers grains (DDG), the main by-product of corn ethanol. Fast pyrolysis is an alternative thermal conversion technology for processing biomass. It has recently been optimized to produce a stream rich in levoglucosan, a fermentable glucose precursor for biofuel production. Additional product streams might be of value to the petrochemical industry. However, biomass heterogeneity is known to impact the composition of pyrolytic product streams, as a complex mixture of aromatic compounds is recovered with the sugars, interfering with subsequent fermentation. The present study investigates the feasibility of fast pyrolysis to produce fermentable pyrolytic glucose from two abundant lignocellulosic biomass sources in Ontario, switchgrass (potential energy crop) and corn cobs (by-product of corn industry).

**Results:**

Demineralization of biomass removes catalytic centers and increases the levoglucosan yield during pyrolysis. The ash content of biomass was significantly decreased by 82–90% in corn cobs when demineralized with acetic or nitric acid, respectively. In switchgrass, a reduction of only 50% for both acids could be achieved. Conversely, levoglucosan production increased 9- and 14-fold in corn cobs when rinsed with acetic and nitric acid, respectively, and increased 11-fold in switchgrass regardless of the acid used. After pyrolysis, different configurations for upgrading the pyrolytic sugars were assessed and the presence of potentially inhibitory compounds was approximated at each step as double integral of the UV spectrum signal of an HPLC assay. The results showed that water extraction followed by acid hydrolysis and solvent extraction was the best upgrading strategy. Ethanol yields achieved based on initial cellulose fraction were 27.8% in switchgrass and 27.0% in corn cobs.

**Conclusions:**

This study demonstrates that ethanol production from switchgrass and corn cobs is possible following a combined thermochemical and fermentative biorefinery approach, with ethanol yields comparable to results in conventional pretreatments and fermentation processes. The feedstock-independent fermentation ability can easily be assessed with a simple assay.

## Background

Presently, ethanol production in the United States and Canada is predominately derived from corn grains. The additional utilization of plant residues such as corn cobs or stover can potentially increase the ethanol yield per unit area and utilize existing conversion and distribution infrastructure [1]. Corn cobs were found to yield higher glucose concentrations than other corn residues like stalks or leaves, and are removed from the fields during conventional harvest [2]. As an alternative to food crops, perennial grasses have also been proposed feedstocks for liquid fuels production. Switchgrass (*Panicum virgatum)* is a crop suitable to be grown on marginal lands, and requires less water and nutrients compared to other sources of biomass used in fuel production [3]. However, the common challenge for lignocellulosic biomass is the high recalcitrance to biological conversion technologies and thus the requirement of pretreatment in commercial processes [4]. A multitude of technologies is available with different advantages and disadvantages as recently reviewed elsewhere [5–10]. Fast pyrolysis is commonly used as a tool to increase the energy density of bulky biomass through thermal cracking (400–550 °C in the absence of oxygen); it can alternatively be used as a pretreatment technology combined with biochemical conversion [11–14]. Pyrolysis of biomass typically yields condensable (‘bio-oil’) and non-condensable gases (often used as fuel gas to power the process) and char (‘bio-char’, a possible soil amendment) [15–18]. The composition of the pyrolysis oil and the liquid yield depend heavily on the operating conditions during pyrolysis, as well as the type of biomass used. Liquid yields of up to 75% wt based on biomass intake have been obtained [17]. The most abundant carbohydrate found in pyrolysis oil is levoglucosan, an anhydrosugar which can easily be converted to glucose via acid hydrolysis [19]. Recent studies have focused on ways to increase levoglucosan yields in pyrolytic oils [20] and in its integration to a fermentation processes [12, 13].

Anhydrous sugar yields depend not only on the cellulose content of the biomass, but also on the presence of alkali and alkaline earth metals, which in turn can vary significantly depending on the growth conditions of the plants as well as harvesting time and conditions [21]. Studies have shown that decreasing the presence of these metal ions via mild or strong acid rinsing [22, 23] increases levoglucosan. Yields of 30 and 52 g_levoglucosan_/g_cellulose_ have been achieved when treating the biomass with acid [20, 24]. The most abundant metals present in biomass are magnesium, calcium, sodium and potassium [21]. Even though the effect of these inorganic elements on pyrolysis has been broadly described in several studies [25–28] a detailed and well-established mechanism has not yet been realized. Nevertheless, studies have shown that metals catalyze cellulose depolymerization, and once depolymerized, further catalyze the decomposition of anhydrous sugars. This effect translates into changes in the composition and yield of pyrolytic oils as water and char generation is enhanced [27] along with several other molecules such as acids, ketones, aldehydes, furans and phenols [29]. Studies involving the fermentation of biomass pyrolysates have found that these compounds hamper ethanol production by inhibiting the growth of fermentative microorganisms [30, 31]. A complete avoidance of such by-product formation is technically not possible; therefore, detoxification approaches that allow for cleaning of the pyrolysates before fermentation are needed. Possible options are adsorption on activated carbon [32, 33] and polymer matrices such as XAD 4 or XAD 7 [34], overliming [35], air stripping [33] and solvent extractions [12, 33, 36]. Studies have also shown that possible combinations of these detoxification routes render a cleaner extract [36].

In a previous study, using a pyrolysis-based biorefinery approach, pyrolytic oil from demineralized pinewood was utilized to prepare fully fermentable pyrolytic sugar [12]. Pyrolytic oils were detoxified via water and solvent extraction followed by acid hydrolysis. The growth and ethanol production kinetics were determined via non-linear regression analysis of online process data, allowing to quantify residual inhibitory effects of by-products in the pyrolytic sugars. Ethanol yields based on glucose available during the fermentation step reached 96% of the theoretical value. However, not all initially present glucan was converted to glucose, hence the overall ethanol yield was 41.3% of the maximum theoretical value assuming all glucan in the initial biomass to be converted to ethanol [12]. However, only one source of biomass was tested, and no attempt was made to correlate inhibition to the presence of inhibitors.

**Fig. 1:**
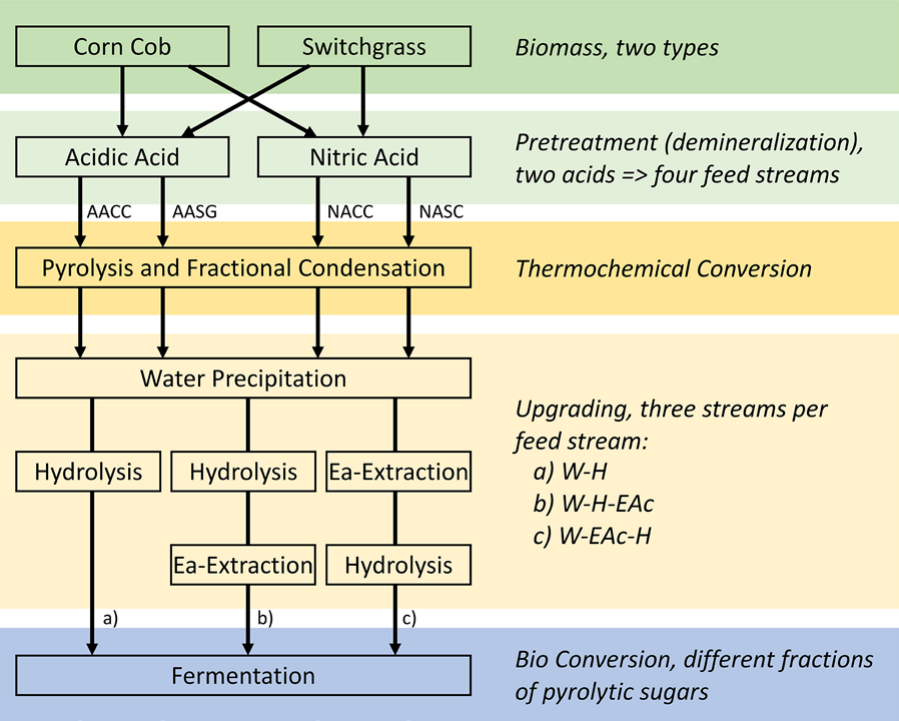
Process schematic for the production of sugars via fast pyrolysis followed by upgrading and yeast fermentation. The feed streams are abbreviated as AACC and AASG for acidic acid-pretreated corn cobs and switchgrass, and NACC/NASG for nitric acid-pretreated corn cobs and switchgrass. The detoxification routes are abbreviated as *a*) W-H: cold water extraction followed by hydrolysis; *b*) W-H-EAc: cold water extraction followed by hydrolysis and ethyl acetate extraction; and *c*) W-EAc-H, cold water extraction followed by ethyl acetate extraction and hydrolysis

The objective of this study is to evaluate the production of ethanol using the pyrolysis-based biorefinery approach (Fig. 1) from two underutilized biomasses in Canada, corn cobs and switchgrass. Two demineralization steps were evaluated to determine how removal of alkaline ions from biomass affects ethanol yields. Furthermore, a simple HPLC assay was developed to estimate the sugar to inhibitor ratio, which was subsequently used as a substrate-independent indicator for fermentation ability.

## Results and discussion

The biorefinery approach depicted in Fig. 1 commences with a demineralization step (two acids were tested), followed by thermochemical conversion via pyrolysis to create a sugar-rich stream for bioconversion. The demineralization reduces side reactions during pyrolysis and favors depolymerization of cellulose. Fractional distillation of the pyrolysis product was used to obtain a ‘bio-oil’ rich in the anhydrous sugar levoglucosan. These oils were upgraded though various combinations of water and solvent extraction to remove by-products, and acid hydrolysis to convert levoglucosan to glucose (Fig. 1).

### Effects of demineralization

Metals such as Ca, K, Mg and Na, occur intrinsically in plant biomass. However, these metal ions are known to form catalytic centers during pyrolysis and catalyze biomass decomposition beyond desirable intermediates such as levoglucosan, a glucose precursor [23]. Levoglucosan can be subjected to strong acid hydrolysis, producing glucose, which is the preferred carbon source for fermentative microorganisms. To maximize levoglucosan yields it is, therefore, desirable to have low ion concentrations in feedstocks prior to pyrolysis. Acetic and nitric acid (weak and strong acid) solutions were used to reduce the ion content in both corn cobs and switchgrass. The initial ash content of the switchgrass used in this study was 40 and 27.9 g/kg for the corn cobs. Ash content in switchgrass can vary between 37.0 [37] and 57.3 g/kg [38] and in corn cobs between 24.1 [39] and 80.6 g/kg [40], thus the measured values are within the typical range. The acid-catalyzed biomass demineralization was more pronounced in corn cobs than it was in switchgrass (Table 1). Post-rinsing ash contents for switchgrass decreased to 55.5 and 54.25% of the original value (40.00 g/kg) after acetic acid and nitric acid washing, respectively; contrasting with the values obtained with corn cobs, 18.2 and 10.2% of the original value (27.90 g/kg). One explanation for the difference in post-rinsing ash content is remaining soil traces from the harvesting process. Despite the higher decrease in the ash content for corn cobs, the alkali content in the demineralized biomass is higher in switchgrass (2.03 and 0.83 g/kg) than in corn cobs (0.85 and 0.47 g/kg), with the majority of these percentages corresponding to different ions, Ca^2+^ in switchgrass and K^+^ in corn cobs, Table 1.

**Table 1:** Metal ions in biomass before and after demineralization and the respective levoglucosan yields

Ion [g/kg]	Switchgrass			Corn Cobs		
	Untreated	Acetic acid	Nitric acid	Untreated	Acetic acid	Nitric acid
Ca^2+^ [g/kg]	2.52 ± 0.20	1.94 ± 0.02	0.76 ± 0.03	0.47 ± 0.03	0.17 ± 0.06	0.06 ± 0.03
K^+^ [g/kg]	11.03 ± 0.20	0.07 ± 0.01	0.05 ± 0.01	15.52 ± 1.47	0.58 ± 0.03	0.34 ± 0.01
Mg^2+^ [g/kg]	0.95 ± 0.06	0.01 ± 0.00	0.01 ± 0.01	0.71 ± 0.01	0.08 ± 0.06	0.04 ± 0.02
Na^+^ [g/kg]	0.09 ± 0.03	0.01 ± 0.00	0.02 ± 0.00	0.07 ± 0.01	0.02 ± 0.00	0.04 ± 0.00
Alkali [g/kg biomass]	14.59	2.03	0.83	16.77	0.85	0.47
Ash [g/kg biomass]	40.00	22.20	21.07	27.90	5.09	2.84
Alkali in ash [%]	36.48	9.15	3.96	60.12	16.68	16.50
Levoglucosan [g/L]	1.39	22.42	23.06	2.16	18.06	28.78
Yield $\left[ \frac{\text{mol levoglucosan}}{\text{mol glucan}} \right]$	0.02	0.30	0.31	0.03	0.23	0.37

Levoglucosan concentrations were obtained after water extraction; the yields are expressed as mole levoglucosan per mole of glucan of the respective biomass (38.80 wt% in corn cobs [66] and 37.00 wt% in switchgrass [67])

Alkaline metal ions such as Ca^2+^ and Mg^2+^ have been reported to catalyze cellulose dehydration and decomposition reactions, whereas ions such as K^+^ and Na^+^ catalyze further degradation of monomeric sugars derived from cellulose [41]. Therefore, the presence of K^+^ and Na^+^ can significantly reduce the yield of levoglucosan [42], and diverts the reaction towards the production of lighter molecules such as hydroxyacetaldehyde, acetol, formic and acetic acid [43]. In addition to the low levoglucosan yields, formation of these undesirable light products typically affects downstream ethanol production, by hindering the growth of fermentative microorganisms [12].

The effects of biomass demineralization on anhydrous sugar production are shown in Table 1. Levoglucosan production from corn cobs increased ninefold with acetic acid pretreatment, compared to a 14-fold increase if pretreated with nitric acid. This increase in production is the result of decreasing the ash content from 5.09 to 2.84 g/kg when nitric acid is used as a rinsing agent in corn cobs. Strong acids such as nitric acid are more effective in removing ions such as Ca^2+^ [44] as evidenced in Table 1. Previous studies have linked Ca^2+^ with increased cellulose thermal stability [45], likely explaining the observed levoglucosan increase when biomass was pretreated with nitric acid. These increases in levoglucosan concentration after mineral removal are higher than previous results where pinewood demineralization was responsible for increasing levoglucosan by a factor of six [12]. The increasing molar yield shows that the levoglucosan is being diverted away from cracking reactions which would create lighter molecules and possible fermentation inhibitors. Nevertheless, molar yields could be further improved by tailoring demineralization to each biomass. These marked contrasts in anhydrous sugar production from different types of biomass, pretreated under the same conditions, can be due to the different biomass compositions and how the pretreatments affects each one directly, as it is known that biomass composition plays a key role in the product’s profile of pyrolysis [17].

### Pyrolysis oil upgrading

Conversion of levoglucosan to glucose, and further purification of the sugar-rich stream was necessary for fermentative conversion. To remove insoluble lignin and hydrophobic inhibitory compounds, all the oils were subjected to a cold water extraction (W) [46], which is the first step in the upgrading of the pyrolytic oils, Fig. 1. Three detoxification approaches were studied. The first approach comprised acid hydrolyzing the levoglucosan in the water extracts to glucose, followed by a neutralization step (W-H, stream a in Fig. 1). The second approach was identical but included a solvent extraction using ethyl acetate (W-H-EAc, stream b in Fig. 1) after the hydrolysis. This step was chosen to remove inhibitory compounds which remained after the water extraction and also that were generated as a result of the strong acid hydrolysis, as it has been widely documented [47–49]. The third approach consisted of cold water extraction directly followed by solvent extraction prior to strong acid hydrolysis and neutralization (W-EAc-H, stream c in Fig. 1). Glucose production from levoglucosan hydrolysis does not appear to be substantially affected by any of the detoxification routes nor by the type of acid used as seen in Table 2. However, it is important to note that no statistical evaluation of these data was performed due to the small amount of starting material available. Nevertheless, these results contrast with findings on pinewood pyrolysates [12], where glucose molar yield was lower, 0.88, but the final glucose concentration was higher 41 g/L. The observed fluctuations are likely a result of residual cellobiose or other oligomers that are also being hydrolyzed to glucose, a known effect that can result in molar yield (glucose per levoglucosan) >1 [50].

**Table 2:** Carbohydrate concentrations and molar yields after each detoxification approach

Biomass	Ion removal type	W-H			W-EAc-H			W-H-EAc		
		Levoglucosan (g/L)	Glucose (g/L)	Molar yield	Levoglucosan (g/L)	Glucose (g/L)	Molar yield	Levoglucosan (g/L)	Glucose (g/L)	Molar yield
Corn cobs	Acetic acid	2.08	18.58	1.05	2.20	17.10	0.97	2.20	19.09	1.08
	Nitric acid	1.30	28.41	0.93	1.01	29.07	0.94	0.94	28.27	0.91
Switchgrass	Acetic acid	1.43	26.62	1.14	1.10	26.54	1.12	1.09	27.54	1.16
	Nitric acid	1.16	26.15	1.07	1.05	26.83	1.10	1.17	26.55	1.09

Glucose yields of up to 216% from pyrolysate hydrolysis have been previously reported [49]. The difference between the values obtained by Bennett et al. [49] and the ones obtained in this study could be due to extra anhydrous carbohydrate oligomers not decomposed in the pyrolysis oil used in that study. Bennett et al. [49] reported increasing glucose levels after levoglucosan depletion (20 min) in the hydrolysis step.

**Fig. 2:**
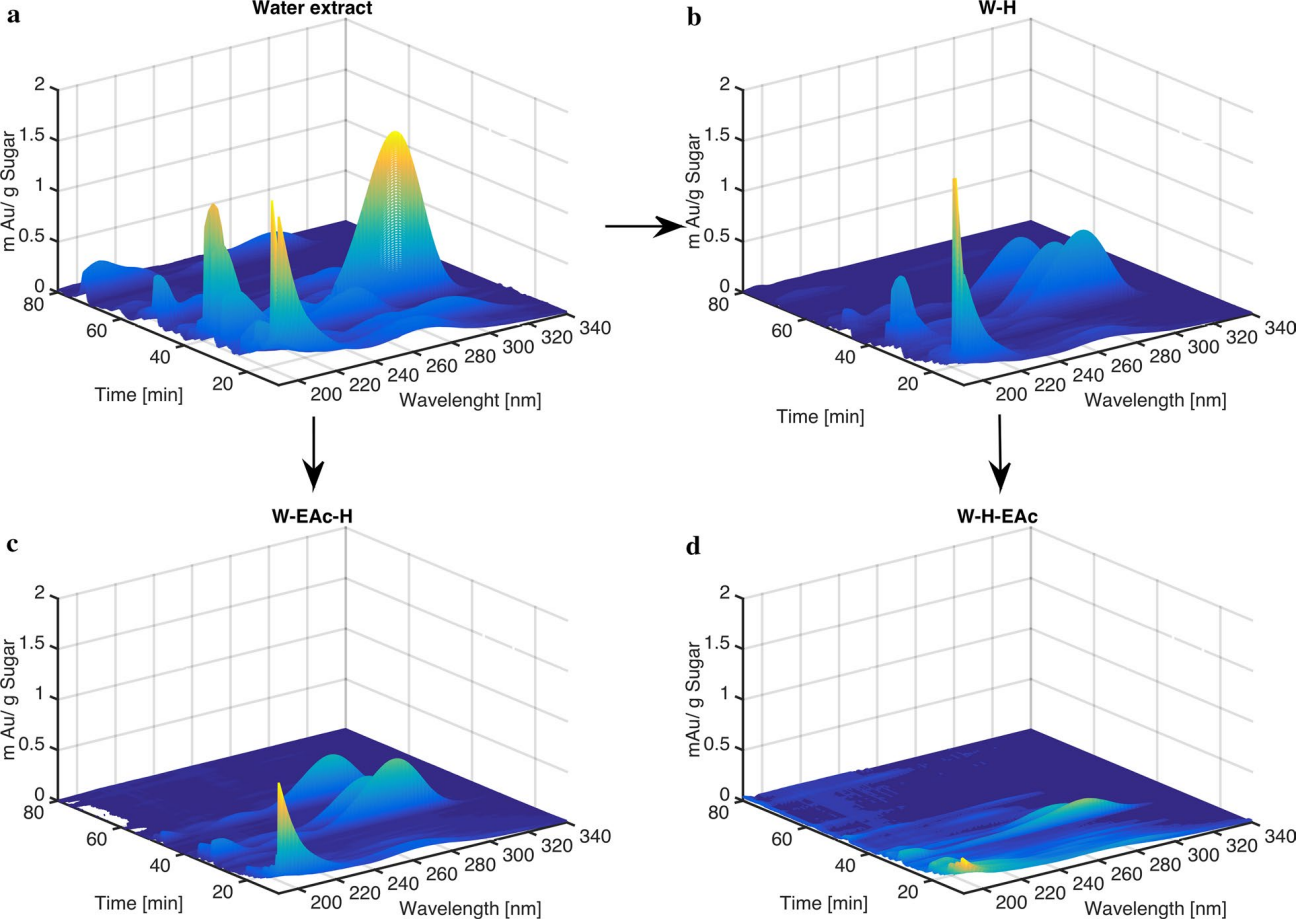
Chromatograms as a function of the different detoxification steps. The extract shown corresponds to NACC pyrolysis oil upgrading. The *arrows* indicate the starting point and the order followed in the process

Typical by-products of the pyrolysis process that tend to inhibit subsequent fermentation are phenols, furans and aldehydes [12, 13, 48, 51]. The cocktail of these compounds is typically very complex and challenging to fully analyze [51–55]. To the author’s knowledge a complete characterization (closed carbon balanced) of a pyrolysis product from lignocellulosic biomass has not yet been accomplished. Feedstock variability would also be expected to change to product distribution from biomass to biomass and likely from batch to batch. A full chemical characterization is hence not suitable if the purpose of the pyrolysis is biofuel production. Many of the possible by-products typically associated with inhibitory effect on fermentation contain chromophores and can hence be detected in the UV range, where carbohydrates do not show a strong signal. A diode array detector (DAD) was, therefore, used to record the chromatogram of the pyrolytic sugar samples between 190 and 340 nm during HPLC analysis of the glucose/levoglucosan concentration (quantified via RID). The relative abundance of peaks is an indication of the residual amount of chromophore-containing by-products. Selected chromatograms after various detoxification steps can be seen in Fig. 2.

The peaks shown in Fig. 2 do not represent the total number of compounds found in the mixtures, and separating peaks (in the time dimension) by varying the HPLC conditions was not attempted. The multiple wavelengths give additional resolution; nevertheless, it is very likely that compounds are co-eluding with the given protocol. However, it can be seen clearly that the upgrading steps remove chromophore compounds. The W-H-EAc sequence results in the cleanest samples (Fig. 2d), likely due to the fact that acid hydrolysis, when performed after solvent extraction (Fig. 2c), produces its own degradation by-products. The volume under the surface shown in Fig. 2 was numerically integrated to obtain a single numerical value and normalized by the sugar (glucose or levoglucosan) concentration in the sample. The value was termed IV/G (integration value over glucose concentration).

1 $${\text{IV/G}} = \mathop \smallint \nolimits_{{t = 10{ \hbox{min} }}}^{{t = 80{ \hbox{min} }}} \mathop \smallint \nolimits_{{\lambda = 190{\text{nm}}}}^{{\lambda = 340{\text{nm}}}} S_{\text{DAD}} {\text{d}}t {\text{d}}\lambda /C_{\text{G}}$$

where IV/G is the glucose normalized inhibitor value, *t* the retention time on the HPLC [min], *λ* the wavelength of the DAD at time *t* [nm], *S*_DAD_ the signal measured at time t and wavelength *λ*, and *C*_G_ the concentration of glucose in the sample [g/L].

**Fig. 3:**
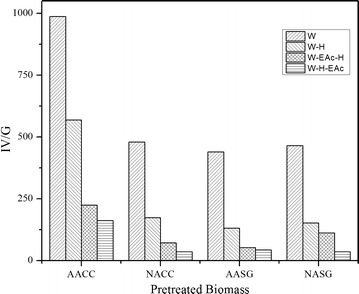
IV/G values estimated for each pyrolytic sugar after the respective upgrading step. Nomenclature for sugar streams and upgrading levels are found in Fig. 1

Figure 3 shows IV/G values for the four different pyrolysates at the various upgrading steps. As expected for all the pyrolytic oils, water extracts, the first step in the upgrading train, showed the highest IV/G. Out of the four water extracts, acetic acid-pretreated corn cobs (AACC) extracts showed the highest IV/G. AACC water extract levels are double or more if compared to nitric acid-pretreated corn cobs (NACC), acetic acid-pretreated switchgrass (AASG) and nitric acid-pretreated switchgrass (NASG) after each detoxification approach (Fig. 3). This high IV/G could be linked to a higher K^+^ presence in the biomass before hydrolysis as shown in Table 1. For all the samples, the steepest decrease was observed after hydrolysis. This reduction can be a result of further decomposition during the hydrolysis step, or through removal during the subsequent Ba(OH)_2_ treatment (added to increase the pH). These findings are in agreement with previous reports where a drop in the total carbon levels was observed when water extracts were neutralized after acid hydrolysis [12]. Conversely, the lowest levels of all the samples was observed when EAc extraction was done to previously hydrolyzed and neutralized samples (W-H-EAc), see Fig. 3.

Performing solvent extraction after the hydrolysis steps helps remove non-sugar compounds that survived the hydrolysis/neutralization step, or that could have been generated while in the process. The numerical IV/G value of a given pyrolytic sugar mixture can be useful when evaluating its fermentability.

### Pyrolytic sugar bioconversion

**Fig. 4:**
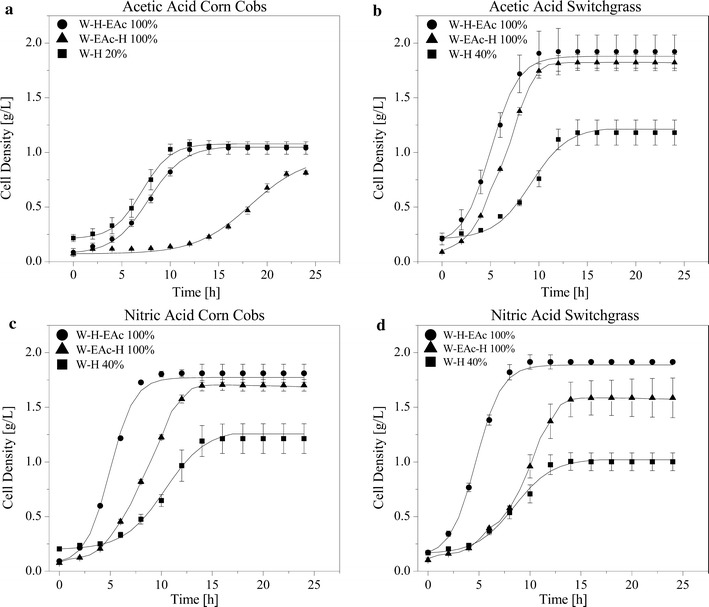
Growth profiles corresponding to the highest pyrolytic sugar fractions (highest IV/G values) where growth was achieved for each of the extracts tested. The initial sugar concentration was 25 g/L for all the blends tested. The percentages in the legends represent the fraction of pyrolytic sugar at the beginning of the fermentation. The *solid lines* represent the best fit of the Baranyi model, while the discrete data points show the average of four replicate fermentations. *Data points* are shown in 2-min intervals, for visual clarity, while data were recorded every 10 s

Micro-scale fermentation experiments were conducted to evaluate the pyrolytic oil extracts as fermentation substrates, and to validate the IV/G value as an indicator for possible inhibitory effects caused by the impurities. The total initial glucose concentration was set to 25 g/L and fermentation broths with various IV/G values were achieved by blending the pyrolytic oil extracts (pH adjusted to 6.5) with a glucose stock solution [12]. In doing so, a range between 20 and 100% of pyrolytic glucose in the fermentable media was achieved. By having different fractions of pyrolytic sugar, proportional fractions of unremoved non-sugar compounds (represented by the IV/G value) were also present, thus enabling the determination of tolerance and threshold levels of *S. cerevisiae* to these compounds [36, 47]. Growth curves of *S. cerevisiae* on pure pyrolytic sugars are shown in Fig. 4. Growth profiles for water extractions only (W-H) showed the strongest inhibition effects. No growth was observed for blends above 60% pyrolytic sugars in any of the biomass extracts tested. The highest tolerance in AACC W-H extracts was at 20% of pyrolytic sugars and 40% pyrolytic sugars for NACC W-H, AASG W-H, and NASG W-H. Similarly, strong inhibition was also observed with pinewood hydrolyzed water extracts as reported elsewhere [12] and confirms that cold water extraction of the pyrolytic oils fails to extract sufficient quantities of inhibition compounds. Nevertheless, growth on 100% pyrolytic sugars was observed when a solvent extraction (W-EAc-H and W-H-EAc) was performed (Fig. 4), with growth being favored when solvent extraction was the last step in the upgrading train (W-H-EAc).

### Kinetic evaluation

Measured growth data were fitted to the Barnayi model via least squares regression (MATLAB, Mathworks Inc). The model consists of two differential Eqs. (2, 3) and three parameters; *µ*_max_ (maximum growth rate), *λ* (adaptation time) and *N*_max_ (maximum biomass density). *Q* is an adjusting function and *Q*_0_ can be seen as parameter representing the initial adaptation of the yeast to new cultivating conditions.

2 $$\frac{{{\text{d}}N}}{{{\text{d}}t}} = \mu_{ \hbox{max} } \left( {\frac{Q}{1 + Q}} \right)\left( {1 - \frac{N}{{N_{ \hbox{max} } }}} \right)N$$

3 $$\frac{{{\text{d}}Q}}{{{\text{d}}t}} = \mu_{ \hbox{max} } Q$$

4 $$\lambda = \frac{{{ \ln }\left( {1 + \frac{1}{{Q_{0} }}} \right)}}{{\mu_{ \hbox{max} } }}$$

**Fig. 5:**
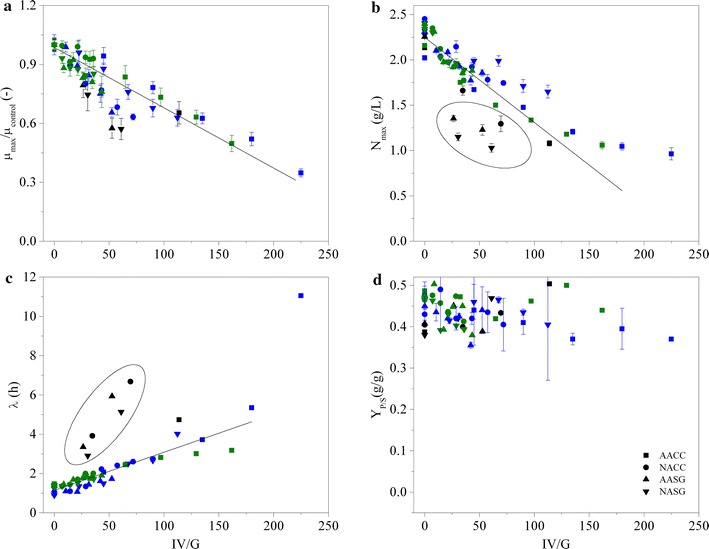
Estimated model parameters for fermentation experiments with varying fractions of unremoved inhibitory compounds resulting from the pyrolytic oils, **(a**–**c**). **d** Ethanol yields from each of the fermentation experiments. The *colors* represent a specific detoxification route, symbols shown in *black* represent samples from detoxification step (W-H), *blue* represents W-EAc-H while *green* represents W-H-EAc. The *X-axis* shows the relative amount of inhibitory compounds (IV/G)/µL in the total volume of the micro-fermentations. AACC stands for acetic acid corn cobs extracts, ANCC nitric acid corn cobs extracts, AASG for acetic acid switchgrass and NASG for nitric acid switchgrass. The *solid lines* represent linear regression analysis of all data with IV/G > 200. The *data points* in the *circles* were excluded from the regression analysis

The respective best fits are depicted by solid lines for the selected data shown in Fig. 4. It can be seen that the Baranyi model adequately describes the data; hence, the numerical values of the model parameters can be used to quantify the effect of unremoved impurities in the pyrolytic sugar as previously described [51]. The correlations between IV/G values and each of the estimated model parameters and measured ethanol yields are shown in Fig. 5. IV/G is negatively correlated with *µ*_max_ and *N*_max_, while it is positively correlated with *λ* and not correlated with Y_P/S_ (ethanol yield). The parameter estimates are plotted as a function of the IV/G value of each micro-fermentation, which varied based on the biomass sources (type of symbol) as well as the level of upgrading (color of symbol). Additionally, the different blends of each pyrolytic sugar result in further variation of the IV/G value (same symbol and color). The distribution and compositions of impurities in the pyrolytic extracts differs for each pyrolytic sugar stream, and the IV/G value is only an approximation of the total amount of impurities.

The observed decrease of *µ*_max_ is a common response of microorganisms subjected to environmental stress. The data for *µ*_max_ are strongly correlated with IV/G, independent of the sugar source. Linear regression analysis was conducted based on all available data points for the maximum growth rate (solid line in Fig. 5a), leading to Eq. 5:

5 $$\mu_{ \hbox{max} } \; = \;0.9859\; \pm \;0.0134\; - \;\left( {0.0031\; \pm \;0.0004} \right)\; \times \;{\text{IV/G}},\;{\text{adj}}.\;R^{2} \; = \;0.672$$

**Fig. 6:**
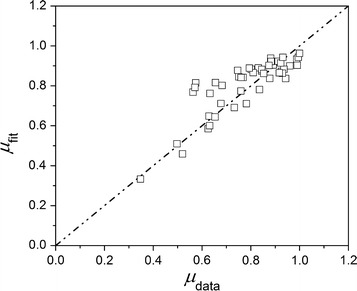
Parity plot of *µ*_max_ directly estimated from growth profiles versus the predicted *µ*_max_ based on the correlation shown in Eq. (5) and measured IV/G value

A parity plot based on Eq. 5 is given in Fig. 6 highlighting the correlation between increased IV/G values and the kinetic parameter. The correlation is improved over a model proposed by Wood and collaborators [51], where different defined inhibitory cocktails were used in a central composite design experiment to determine significant factors, and a model based on the concentrations of known inhibitory concentrations was proposed. However, Wood’s model requires the knowledge of the concentrations of six specific inhibitors, and has only been tested over a well-defined range of concentration of these compounds with growth medium otherwise free of impurities [51], while the IV/G model only requires a single parameter.

The data are more spread for the parameters *λ* and *N*_max._ The response of these parameters appears to be more strongly affected by the composition of the cocktails than the maximum specific growth rate. Particularly sugars that have only being upgraded via water extraction and hydrolysis (black symbols in Fig. 5) appear to exhibit longer adaptation phases (*λ*) than samples subjected to solvent extraction (blue and green symbols) with the same IV/G value. The remaining data are linearly correlated for IV/G <200:

6 $$\lambda \; = \; 1.4423\; \pm \;0.0755\; + \;\left( {0.0194\; \pm \;0.00123} \right)\; \times \;{\text{IV/G, adj}}. R^{2} \; = \;0.8367$$

Similarly, the maximum cell concentration achieved during fermentation decreased most in samples subjected to water extraction only (black), where the switchgrass-derived sugars (triangles) were affected particularly strongly. The general decrease of the final cell concentrations with increasing IV/G values appear to be a logical consequence of inhibition and a linear correlation can be found in the data in Fig. 5b (the circled data points were not considered for the regression analysis):

7 $$N_{ \hbox{max} } = { 2}.2481 { } \pm \, 0.0281 { } + \, \left( {0.0094 { } \pm \, 0.0008} \right)\; \times \;{\text{IV/G}},{\text{adj}}. \, R^{2} = \, 0.6996$$

The total amount of ethanol produced does not appear to be affected by the presence of otherwise inhibiting compounds. The ethanol yield was unaffected for the chosen micro-fermentations. However, the ethanol yields shown in Fig. 5d are all based on micro-fermentations where cell growth was observed. The final yeast concentration was always >1 g/L (Fig. 5b) with growth rates >30% of the uninhibited growth (Fig. 5c). At higher IV/G values, either no cell growth was observed, or limited cell growth was not suitable to estimate kinetic parameters of the Barnayi model. In such cases, no ethanol yield was determined.

The data clearly show that complex inhibitory cocktails affect microbial growth kinetics in a multitude of ways, with some aspects of the yeast’s growth being more sensitive to the composition of the impurity mix (*λ* and *N*_max_) than others. A simply estimate of the inhibitory potential of pyrolytic sugars can be made based on the proposed parameter IV/G, particularly for the maximum specific growth rate. The maximum specific growth rate is arguably the most important parameter, as the overall ethanol yield was not affected over the observed range (for datasets where sigmoidal growth patterns were observed).

The increased adaptation phase can likely be addressed through acclimation of the inoculum. Consequently, a better adapted inoculum might also help increase final yeast concentrations. The observed results are in agreement with previously reported data on pinewood pyrolysate [12], as is the fact that the ethanol yield was not affected by the inhibitors, which has also been shown before for furans and phenols [56].

The correlation based on IV/G values (Eqs. 5–7) appear to be capable of predicting effectively the synergistic effects of different compounds found in the pyrolytic oil. The applicability of the IV/G value beyond a single type of biomass and a single pretreatment and upgrading is highly relevant when screening for possible biomass sources, and possibly gives this parameter a general meaning beyond this specific study. It should be noted that IV/G values are limited by the resolution of the HPLC methodology. Hence, it could prove useful to have an extended method development step resolving more compounds. By doing so, a stronger correlation might be achieved by the model, while is also the possibility that non-inhibitory compounds could be resolved and their presence in the chromatogram might result in weaker correlations; however, this was beyond the scope of this study.

### Ethanol production

The reported ethanol yield was solely based on glucose consumption. Possible ethanol production from other sugars was not considered even though they can be present after pyrolysis and hydrolysis [13]. The maximum yield achieved was 0.49, corresponding to a 96% of the theoretical maximum. These results are in agreement with previous studies performed on pyrolysates pinewood [12], where the authors suggested that a possible diversion of the carbon flux from yeast (biomass) to ethanol might occur. Other studies suggest that an increasing amount of acetate triggers a rise in ATP requirement levels [48] which is linked to higher ethanol titers under anaerobic conditions. Samples for ethanol analysis were taken 2 h after reaching a stationary phase, securing a depletion of glucose and avoiding any possible ethanol loss due to evaporation. Ethanol production was achieved at the highest concentrations of total inhibitors still allowing for cell growth, Fig. 5d.

**Fig. 7:**
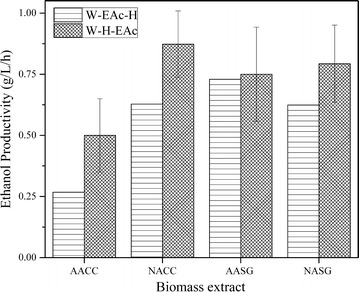
Ethanol productivity for fermentation samples with the highest concentration of total inhibitors (blends with 100% of pyrolysis-derived sugar)

Another important feedstock characteristic is the ethanol productivity (rate) [56]. The ethanol productivity was defined as the amount of ethanol produced by the cells divided by the time at which they reached stationary phase (relative change in OD_600 nm_ < 0.025 OD/h). Figure 7 shows the effect of pretreatment and upgrading on ethanol productivity. EAc extraction after the hydrolysis is responsible for the increases seen in three of the four biomass extracts used. AACC ethanol productivity increased from 0.27 to 0.5 g/L/h, NACC from 0.63 to 0.88 g/L/h and NASG 0.62 to 0.8 g/L/h, each corresponding to 85, 40 and 30% increases, respectively. These increases in productivity are connected to the total content of inhibitors, which is reduced if EAc extraction is conducted after the hydrolysis (Fig. 2). The estimated productivities are largely useful as relative values within this study and cannot be directly compared with typically higher values reported in the literature [57], due to the scale and setup of the experimental system (micro-scale, non-optimized seed culture, etc.).

Most previous studies only investigated the effects of single inhibitory compounds on ethanol productivity, such as ferulic acid, 4-hydroxycinnamic acid [58] syringic acid [57, 59, 60] among others. In this study, the hydrolysate is considered as a whole inhibitory unit accounting for overall synergistic effects between the produced compounds.

The total amount of ethanol produced per 100 g biomass was between 3.2 and 6.2 g for corn cobs, between 5.4 and 5.7 g for switchgrass (Table 3), corresponding to 14.6 to 27.8% and 25.7 to 27% of the theoretical maximum yield (assuming the full conversion of all glucan to ethanol). These values are lower than what has been reported for pinewood (8.2 g ethanol, 41.2% of the theoretical yield). The difference between the ethanol yields is likely a result of the type of biomass. Even though pinewood has a lower cellulose content than corn cobs and switchgrass, 35 vs 38.8 wt% and 37.0 wt%, respectively, carbon was mainly lost in the pyrolysis process, the levoglucosan yield after pyrolysis was higher for pinewood 0.51 [12], contrasted with 0.23 in corn cobs and 0.30 in switchgrass (Table 1), and is reflected in ethanol yields (Table 3).

**Table 3:** Ethanol mass balances based on 100 g of starting biomass material

Biomass	Detoxification route	Acetic acid		Nitric acid	
		Ethanol (g)	Ethanol % of theoretical	Ethanol (g)	Ethanol % of theoretical
Corn cobs	c)	3.2	14.6	5.9	26.8
	b)	3.6	16.5	6.2	27.8
Switchgrass	c)	5.7	27.0	5.6	26.8
	b)	5.5	26.4	5.4	25.7
Pine wood	c)	8.2	41.3	–	–

The pine wood value was previously reported by Luque and collaborators [12]. Detoxification route c) was W-EAc-H and route b) was W-H-EAc

The difference could be due to the ion content, as herbaceous biomasses (e.g., corn cobs and switchgrass) can contain ten times more alkali and alkaline earth metals than softwood biomass such as pinewood, which might translate into a lower levoglucosan yields [61]. Despite the differences observed between the yields reported herein and other well-established lignocellulosic ethanol production processes (between 54 and 85% based on available hexoses [62, 63]), the entailed process is one of the many streams generated in thermochemical biorefinery concept, where valuable products like bio-char and bio-gas are generated in the pyrolysis steps, and where streams branching from the upgrading step, phenols, aldehydes and furans can be used as platform chemicals [29] or as added value products [13].

This study shows that fermentable substrates for ethanol fermentation can be produced from agroindustrial waste biomass, e.g., corn cob and switchgrass, via fast pyrolysis. Optimization of each step was beyond the scope of this study but leaves room for further studies to increase the feasibility of the process.

## Conclusions

This study demonstrated that switchgrass and corncobs showed to be suitable lignocellulosic feedstocks for ethanol production via fast pyrolysis. Biomass demineralization enhanced levoglucosan production and decreased the inhibitors’ concentration in the resulting pyrolytic oils. The normalized inhibitor value (IV/G) proved to be an efficient tool for quantifying the relative presence of the inhibitors, thus rapidly assessing the potential for a pyrolytic oil to be a source of fermentable sugars. A simple extraction reduced the inhibitor fraction enhancing ethanol productivity (0.88 g/L/h) while maintaining high ethanol yields (96% of theoretical). Despite the high ethanol yield, it corresponds only to a 28% of the theoretical yield based on the total cellulose available.

## Methods

### Biomass pretreatment and characterization

Once reduced to the required particle size, 1–2 mm, biomass was subjected to demineralization with a weak acid solution (Acetic Acid 10% V/V) or a strong acid solution (HNO_3_ 10% V/V). Biomass was added to the acid solution in a 1:10 ratio (w/V). The mixture was stirred, 1200 rpm, for 2 h at 50 °C in a jacketed vessel to secure proper contact of the biomass with the solution [20]. Once the stirring was completed, the biomass was rinsed by removing the acid solution and adding Milli-Q water (Milli-Q Integral 5, EMD Millipore, USA) in batches of 1 L and stirred for 5 min at room temperature. The final rinsing step was determined by monitoring conductivity (Pinnacle Series, Nova Analytics, USA) of output water stream until the value approached zero and remained constant.

To reduce moisture, rinsed biomass was dried at a 105 °C for 24 h in a convection oven (Thermo Scientific, USA). Final moisture was recorded using a moisture analyzer (ADAM, USA). The ash content of the biomass was determined by quantifying the residue remaining after 24 h of dry oxidation at 575 °C [64]. The ashes were dissolved in 2 wt% sulfuric acid and analyzed by ICP-OES with a radial plasma (Varian Liberty II) for their Na, K, Mg and Ca concentration.

### Anhydrous sugars production

Anhydrous sugars were produced using a biorefinery approach detailed in Fig. 1. Two different oils for each biomass were produced, to compare demineralization approaches and their impact on the pyrolytic oil potential as fermentative substrates for ethanol production.

Batches of 100 g of dried biomass were thermally decomposed in a fluidized bed pyrolyzer at 480 °C with a vapor residence time <2 s. Fractional condensation of vapors was achieved using two condensers in series kept at 1.1 ± 0.01 bar. The fraction recovered in the first condenser set at 80 °C was an oil rich in aromatics and sugars. The second condenser, set at −20 °C, yielded a fraction rich in acetic acid and water. This second condenser liquid is used in the demineralization of the biomass, due to its high acetic acid fraction as detailed elsewhere [29].

### Upgrading

Insoluble lignin was precipitated from the obtained pyrolytic oil samples via cold water extraction [46]. Pyrolytic oil was added to cold water (4 °C) under heavy stirring (900 rpm) in a baffled beaker. Oil was added until the oil to water ratio reached 1:10 (w/w). Insoluble lignin was measured gravimetrically and removed via filtration using a pre-dried and weighed 0.2-µm membrane. Filtrate was collected and stored at 4 °C [12]. Each of the pyrolytic oils followed the same pretreatment method, thus obtaining four different water extracts.

Three different approaches were used to procure the fermentable sugars, Fig. 1. The first consisted of directly hydrolyzing the water extracts to produce glucose, referred as W-H (water extract to hydrolysis). After hydrolysis of the water extract, an additional extraction with ethyl acetate was performed (W-H-EAc). The third approach involved extracting the water extract with ethyl acetate before acid hydrolysis to produce glucose, and referred to as W-EAc-H, and previously reported elsewhere [12].

Solvent extractions aimed to remove organic compounds known to hinder yeast fermentation. A slight modification to the extraction method reported by Luque and collaborators [12] was implemented. All solvent extractions were performed as follows. Ethyl acetate was added to produce a solution with a 1:2 w/w hydrolyzate (depending on the approach taken) to ethyl acetate ratio. The solution was then mixed for 12 h at 150 rpm and 25 °C in a temperature-controlled shaker (Infors, Switzerland). Once mixed, the mixture was transferred to a separating funnel and left to stand for 24 h to ensure proper phase separation. The resulting bottom layer was collected and subjected to evaporation to remove any ethyl acetate residue at 50 °C using the controlled temperature shaker (Infors, Switzerland). The ethyl acetate concentration was monitored by analyzing samples hourly via HPLC until the concentration reached a constant value. The sugar concentration was kept constant by adding water.

Glucose was produced via strong acid hydrolysis of levoglucosan. Extract aliquots of 7 mL were transferred to a microwave vial (VWR, USA), proceeded by the addition of H_2_SO_4_ (Caledon, Canada) to a final concentration of 0.5 M. Vials were sealed and hydrolysis was carried out using an autoclave for 20 min at 120 °C [49]. Hydrolysates were transferred to 15-mL centrifuge tubes (VWR, Canada) and the pH was adjusted to 6.5 by adding Ba(OH)_2_ (Alfa Aesar, USA). Formed crystals were then precipitated via centrifugation at 3500 rpm for 20 min (Sorval ST40R, Thermo Scientific). Supernatants were transferred to new sterile 15-mL centrifuge tubes after filtration (0.2-µm cellulose syringe filter, VWR, Canada).

### Inhibitors removal quantification

Before and after each detoxification step (Fig. 1) samples were analyzed via HPLC (Agilent 1260 series, USA), utilizing a Hiplex H column (Agilent, USA) kept at 60 °C with 5 mM H_2_SO_4_ as the mobile phase at a flow rate of 0.7 mL/min for 80 min. Spectra between 190 and 340 nm were recorded with a 2-nm step utilizing a diode array detector (DAD) (80 Hz). Raw data were exported and processed in MATLAB (MathWorks Inc, USA). The volume under the recorded spectra was numerically integrated in the time and in the wavelength dimension to determine a single value, which was then normalized by the sugar concentration of the sample also determined by HPLC. The inhibitor value IV/G was defined in Eq. 1.

Removal performance was evaluated based on changes in the IV/G value after each detoxification step.

### Fermentation

After the required detoxification steps, yeast extract, peptone and glucose (YPG) media was prepared using the obtained hydrolysates, by adding solid peptone (BD, USA) and yeast extract (BD, USA) to a concentration of 2 and 1 wt%, respectively. Fresh YPG media with the same peptone, yeast extract and regular glucose concentrations (Alfa Aesar, USA) was prepared and blended with the pyrolytic media in different proportions. The high concentrations of pyrolytic glucose obtained in the extracts allowed for a pyrolytic sugar fraction between (20 and 100%). By creating these blends, it was possible to determine the yeast tolerance threshold to unremoved inhibitory compounds dissolved along with the pyrolytic glucose within the media. This method relates to the MIC assay, where an inhibitor is added in increasing concentrations usually correlating this increment to a decrease in cell concentration via turbidity. Here, the method was developed to assess a complex matrix as a whole inhibitory entity.

Blend aliquots of 180 µL were added to microtiter well plates (Costar, Corning USA) and inoculated with previously activated *Saccharomyces cerevisiae* DSM 1334 seed culture (Braunschweig, Germany). Microtiter plates were sealed with a sterile PCR film (VWR, Canada) and punctured with a sterile 18 gauge needle (BD, USA) to allow for gas exchange. Microtiter plates were incubated at 30 °C and 74 rpm for 24 h on a Tecan 200-m microtiter plate reader (Tecan, Austria) equipped with a gas control unit (Tecan, Austria) to secure an anaerobic atmosphere by purging nitrogen throughout the entire process. Optical density, OD_600 nm_, readings were taken of each well by the microplate reader using i-Control software at 10 min intervals to monitor cell density. Glucose and ethanol concentrations were measured at the start and end of the fermentations via high-pressure liquid chromatography fitted with a Hiplex H column at 55 °C, and equipped with an RI detector at 60 °C (Agilent 1260 series, USA). Prepared 0.5 mM H_2_SO_4_ solution was used as the mobile phase and set to flow rate of 0.7 ml/min. End point of each fermentation was defined as two consecutive hours of no absorbance change after reaching the stationary phase.

### Modeling and determination of yeast growth parameters

To calculate inhibition effects on the yeast growth, parameters associated with the growth kinetics were determined by fitting the obtained experimental kinetics data to the model elucidated by Baranyi and Roberts [65]. This model describes cell density as a function of time with three parameters (Eqs. 2–4): *Q*_0_ the initial adaptation of the yeast to the environment, *µ*_max_ the maximum theoretical growth rate and *N*_max_ the maximum value reached by the cell density when the growth kinetics reach the stationary phase. A fourth parameter (Eq. 4), *λ*, corresponding the adaptation time of the yeast to the media was calculated as a function of *Q*_0_ and *µ*_max_.

The differential Eqs. 2 and 3 were solved numerically via MATLAB and least square regression was used to obtain the parameters. The quality of the fit was assessed with normal probability plots. Some important characteristics of this model were explained before [12] as for realizing the adaptation time *λ* to a new media, it uses an adjusting function (*Q*). It is worth noting that the maximum growth rate, *µ*_max_, in this model varies from the one described by kinetics following Monod type behavior, as it is defined as a maximum potential growth rate as opposed to a specific measured value [65].

## Abbreviations

DDG: dried distillers grains
HPLC: high-performance liquid chromatography
W: cold water extraction
EAc: ethyl acetate extraction
H: acid hydrolysis
AACC: acetic acid-pretreated corn cobs
NACC: nitric acid-pretreated corn cobs
AASG: acetic acid-pretreated corn cobs
NASG: nitric acid-pretreated corn cobs
W-H: water extract followed by hydrolysis detoxification route
W-H-EAc: water extract followed by hydrolysis followed by an ethyl acetate solvent extraction
W-EAc-H: water extract followed by ethyl acetate solvent extraction followed by Hydrolysis
YPG: yeast extract peptone glucose
*µ*_max_: maximum growth rate
*λ*: lag time of adaptation
*N*_max_: maximum cell density
Q_0_: parameter describing the initial adaptation of the yeast
DAD: diode array detector
RID: refractive index detector
IV/G: integration value over glucose concentration
ATP: adenosine triphosphate
